# Three-dimensional heads-up system assisted pars plana vitrectomy and subretinal recombinant tissue plasminogen activator injection for submacular hemorrhage

**DOI:** 10.1186/s40662-023-00326-5

**Published:** 2023-03-01

**Authors:** Xinyu Zhao, Qing Zhao, Erqian Wang, Ningning Li, Lihui Meng, Wenfei Zhang, Tan Wang, Youxin Chen, Hanyi Min

**Affiliations:** 1 Department of Ophthalmology, Peking Union Medical College Hospital, Chinese Academy of Medical Sciences, No.1 Shuaifuyuan, Wangfujing, Dongcheng District, Beijing, 100730 China; 2 Key Laboratory of Ocular Fundus Diseases, Chinese Academy of Medical Sciences & Peking Union Medical College, Beijing, China; 3 Department of Operating Room, Peking Union Medical College Hospital, Chinese Academy of Medical Sciences, Beijing, China

**Keywords:** Submacular hemorrhage, Three-dimensional heads-up system, Recombinant tissue plasminogen activator, Safety, Efficacy

## Abstract

**Background:**

To evaluate the outcomes of three-dimensional (3D) heads-up system assisted pars plana vitrectomy (PPV) and subretinal injection of recombinant tissue plasminogen activator (rt-PA) for submacular hemorrhage (SMH).

**Methods:**

Medical records of SMH patients who underwent 3D heads-up system assisted-PPV and subretinal injection of rt-PA from June 2021 to January 2022 were reviewed. The main outcomes included best-corrected visual acuity (BCVA), SMH absorption, and perioperative complications.

**Results:**

We finally included 18 SMH eyes, most of which happened secondary to polypoidal choroidal vasculopathy (PCV) (10, 55.56%), followed by retinal arterial microaneurysm (RAM) (5, 27.78%), traumatic retinopathy (2, 11.11%) and neovascular age-related macular degeneration (nAMD) (1, 5.56%). The greatest linear dimension (GLD) and height of SMH were 6538.17 ± 2533.11 μm and 937.36 ± 420.21 μm, respectively. After an average postoperative follow-up period of 131.06 ± 38.96 days, patients’ BCVA improved significantly from 1.85 ± 0.62 to 1.14 ± 0.82 logMAR (*P* < 0.05). SMH was completely and partially absorbed in 9 (50.00%) and 6 (33.33%) eyes, with no occurrence of iatrogenic retinal break. However, 4 additional PPVs were performed to manage the postoperative SMH and/or vitreous hemorrhage (VH) recurrence (2, 11.11%) and retinal detachment (RD) occurrence (2, 11.11%). Preoperative BCVA was significantly correlated with postoperative BCVA in multiple linear regression analysis (*P* < 0.05), and hemorrhagic pigment epithelial detachment (PED) was significantly correlated with SMH and VH recurrence in univariate binary logistic regression analysis (*P* < 0.05).

**Conclusions:**

The 3D heads-up system assisted-PPV and subretinal injection of rt-PA were efficacious in eliminating SMH and improving visual prognosis with satisfactory safety profile, while caution should be taken for PCV patients with hemorrhagic PED and massive SMH.

## Background

Submacular hemorrhage (SMH) is a severe complication associated with polypoidal choroidal vasculopathy (PCV), retinal arterial microaneurysm (RAM), neovascular age-related macular degeneration (nAMD), and traumatic retinopathy. The blood accumulation between the neurosensory retina and the retinal pigment epithelium (RPE) can cause significant damage to the retina [1, 2]. Without timely therapy, the course of SMH is severe and progressive and often causes irreversible vision loss [3, 4]. Therefore, SMH has gained increasing attention from retinal ophthalmologists.

The intravitreal or subretinal injection of recombinant tissue plasminogen activator (rt-PA) in treating SMH has been evaluated by many studies. The displacement of SMH by subretinal injection of rt-PA combined with pars plana vitrectomy (PPV) and vitreous cavity tamponade showed a promising prognosis and had become the standard therapy for treating SMH patients [5–7]. However, several issues of this novel treatment strategy still need to be settled. First, the previous surgical management was mostly conducted using the traditional microscopic (TM) system. In 2016, Eckardt et al. [8] applied the three-dimensional (3D) heads-up system in complicated posterior segment surgeries, sparking ophthalmologists’ interest in using this system to treat vitreoretinal diseases. The 3D heads-up system was reported to have multiple advantages over the TM system, such as higher magnification, a wider visual field, superior stereoscopic sensation, increased depth of field, improved ergonomic design, and enhanced surgical team communication and education [9–14]. However, the application of the 3D heads-up system in the treatment of SMH has not been evaluated. Second, the best way to reduce the risk of postoperative retinal detachment (RD) and recurrence of SMH and vitreous hemorrhage (VH) remains unknown [5]. Previous studies reported that the risk of these surgery-related complications was high, and sometimes repeated vitrectomies were needed [6, 15]. Third, the proper way to reduce the mechanical damage to the sensory retina and RPE during subretinal injection needs further exploration [2, 16, 17].

Recently, we applied 3D heads-up system assisted-PPV and subretinal rt-PA injection in treating SMH and obtained promising effectiveness and safety. In this study, we reported our experience at a tertiary medical center, providing a reference for ophthalmologists when facing with similar situations.

## Methods

### Study design

This study was a retrospective case series. Medical records of patients who had undergone 3D heads-up system assisted-PPV and subretinal injection of rt-PA for SMH were reviewed. These patients were examined and treated by two surgeons (YXC and HYM) at the Ophthalmology Department of Peking Union Medical College Hospital (PUMCH) in Beijing, China, from June 2021 to January 2022. This retrospective study was approved by the Institutional Review Board/Ethics Committee of PUMCH (No. S-K1993) and was conducted following the tenets of the Declaration of Helsinki. ﻿Written informed consent for the agreement on the detailed operation and the instruments used in the surgery was obtained from all included patients.

### Inclusion and exclusion criteria

The following inclusion criteria were used: (1) Confirmed diagnosis of SMH secondary to PCV, nAMD, RAM, or traumatic retinopathy; (2) Unscarred SMH determined by fundus examination and optical coherence tomography (OCT). The unscarred SMH was defined as the clear accumulation of blood between the neurosensory retina and RPE with a red appearance while the scarred SMH was defined as those that were white and/or fibrous in appearance; (3) Patients who underwent 3D heads-up system assisted-PPV and subretinal injection of rt-PA using the 41-gauge (80 μm) subretinal needle for the treatment of SMH; (4) Patients with detailed medical records and underwent comprehensive ophthalmologic examinations including measurement of best-corrected visual acuity (BCVA) and intraocular pressure (IOP), OCT, and fundus photograph (FP). The exclusion criteria were: (1) Any other concomitant ocular diseases that may confound the results pertaining to SMH, such as hemorrhagic pigment epithelial detachment (PED), subretinal scar formation, and Best disease; (2) Patients with insufficient medical data or lost to follow-up.

### Surgical procedure

**Fig. 1:**
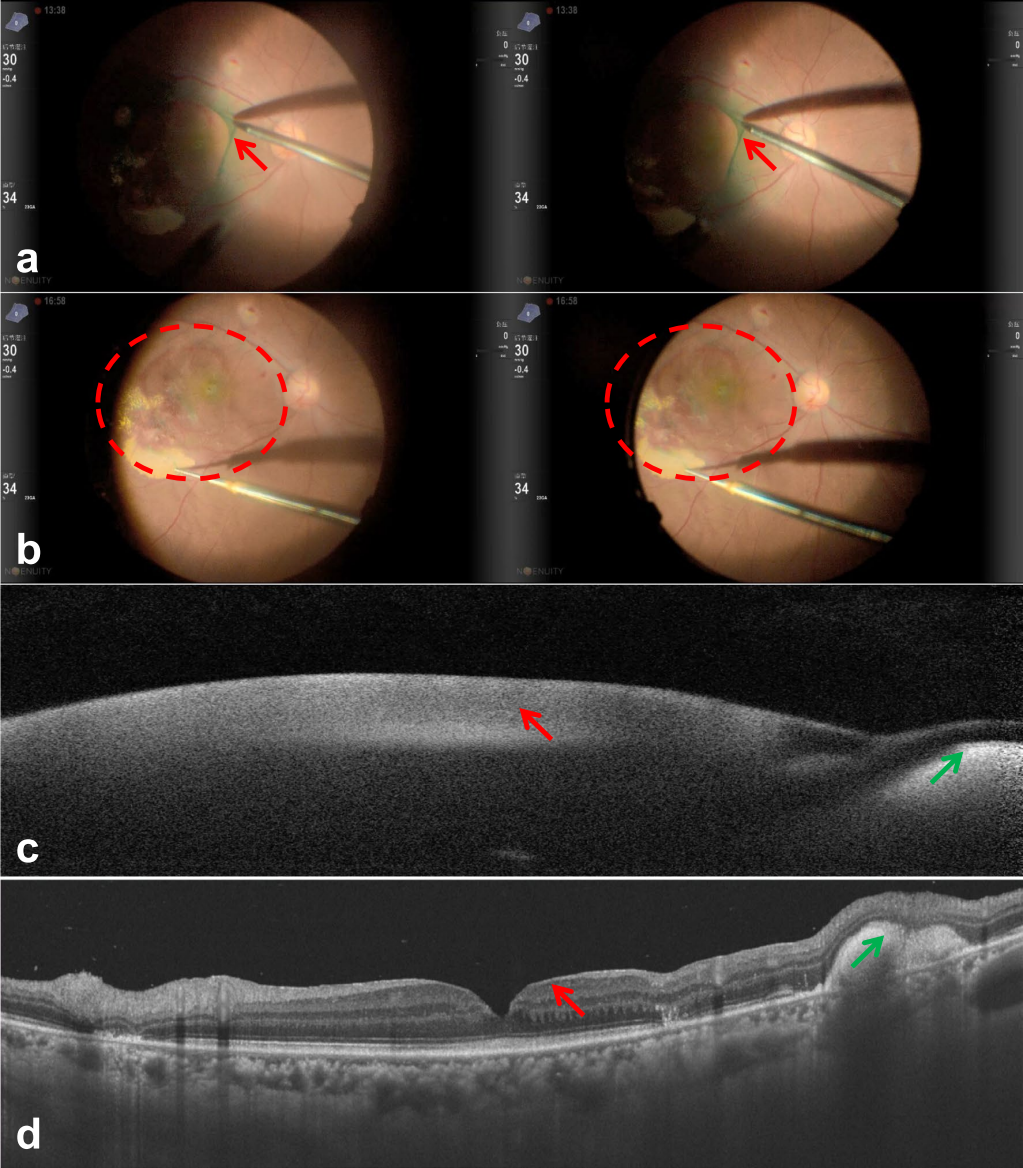
The left eye of a 57-year-old female submacular hemorrhage (SMH) patient secondary to retinal arterial microaneurysm. **a** The three-dimensional (3D) heads-up system assisted internal limiting membrane (ILM) peeling. The 3D effect will be shown if readers wear polarized 3D glasses. Red arrow shows the peeled ILM; **b** Subretinal injection of recombinant tissue plasminogen activator (rt-PA) using the 41-gauge subretinal needle. The 3D effect will be shown if readers wear polarized 3D glasses. The red dashed circle showed the subretinal accumulation of rt-PA and the subretinal bleb around the subretinal hemorrhage; **c** Preoperative swept-source optical coherence tomography (SS-OCT) showed large preretinal hemorrhage (red arrow) and large SMH (green arrow); **d** Postoperative SS-OCT showed complete removal of the preretinal hemorrhage (red arrow) and partial absorption of SMH (green arrow)

All surgeries were performed with the Alcon NGENUITY® 3D Visualization System (Alcon Laboratories, Fort Worth, TX, USA). All patients underwent standard 25-gauge three-port PPV under local retrobulbar anesthesia. After the eyes were disinfected with 5% povidone-iodine, trocar cannulas were inserted at a 20–30° angle into the conventional inferotemporal, superotemporal, and superonasal quadrants 3.5–4 mm posterior to the limbus. Posterior vitreous detachment and vitrectomy were routinely conducted. Then, internal limiting membrane (ILM) peeling, limited by the vascular arcades in the posterior pole, was performed with the assistance of indocyanine green (ICG) staining (shown in Fig. 1a). The rt-PA (Actilyse®, Boehringer Ingelheim Pharma GmbH & Co.KG, Germany) was diluted to 0.25 mg/mL with a balanced salt solution. Assisted by the viscous fluid control system with a constant pressure of 10 mmHg, approximately 0.1 mL of rt-PA was injected subretinally using a 41-gauge subretinal infusion needle (MedOne, Sarasota, Florida, USA) to liquefy the SMH. The rt-PA was injected in the location with enough volume of the subretinal hemorrhage to avoid penetrating the RPE. After the subretinal injection of rt-PA, a subretinal bleb formed around the subretinal hemorrhage (shown in Fig. 1b). Then, fluid-air exchange was performed, and 14% perfluoropropane (C3F8) or silicone oil was injected as the tamponade to flatten the retina and then mobilize, displace, and promote the absorption of the subretinal blood. The patients were then instructed to maintain a facedown position or a sitting position for 5 to 7 days.

### Data collection

Information extracted from the medical records of all included patients was as follows: age, sex, diagnosis, operative eye, treatment before SMH onset, the interval from the SMH onset to the time of the surgery, details of the surgery, duration of specific surgical steps (e.g., ILM peeling), choice of tamponades, pre- and postoperative Snellen BCVA, and perioperative complications. The greatest linear dimension (GLD), height of SMH, and foveal involvement were assessed using the swept-source optical coherence tomography (SS-OCT) images. GLD of SMH was defined as the maximal horizontal distance between the two points where the neurosensory retina bulges inward. The postoperative follow-up was scheduled at approximately 1 week, 1 month, and 3 months, with the evaluation of Snellen BCVA, IOP, FP, OCT, and etc.

### Outcome measures

**Fig. 2:**
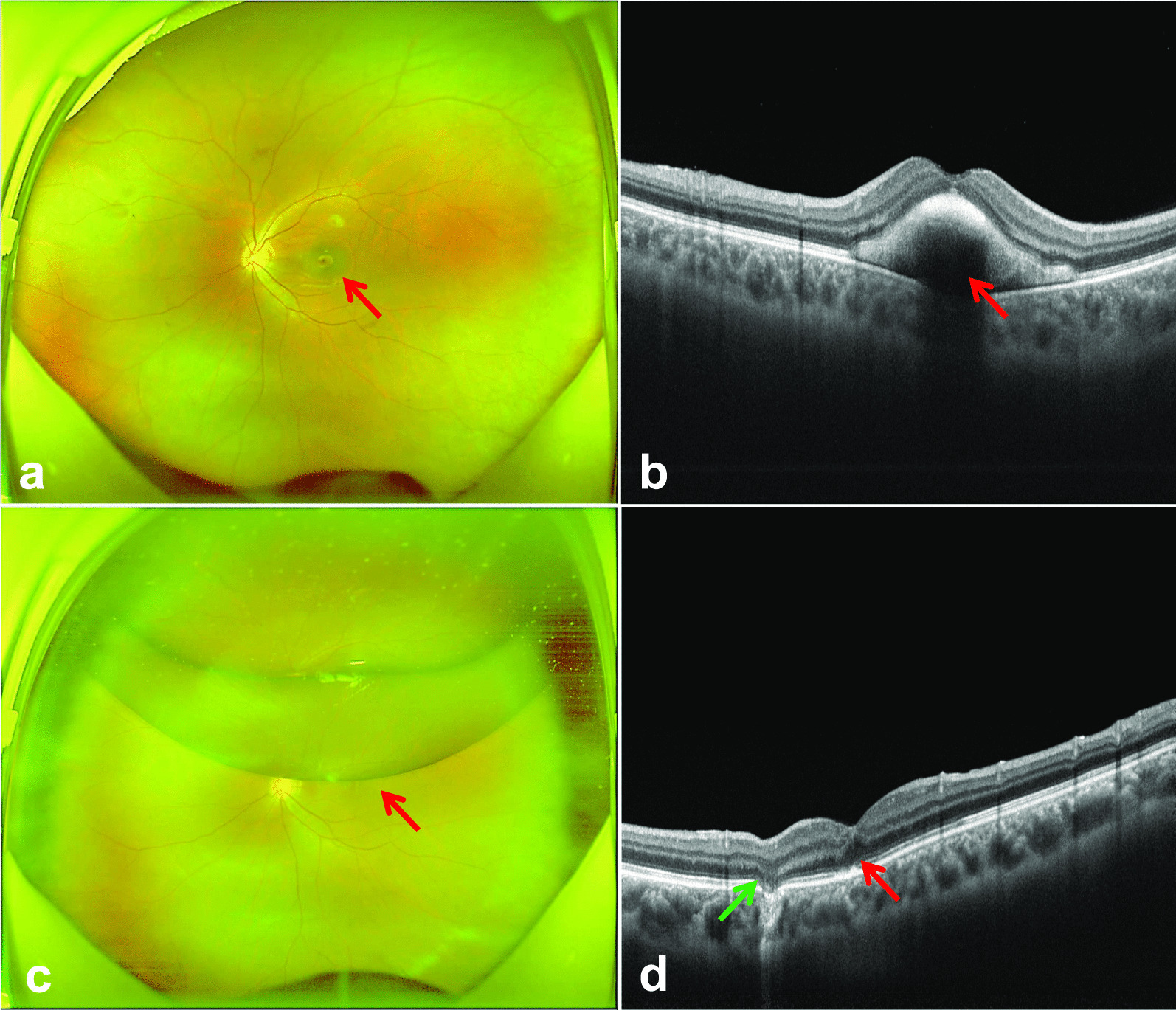
The left eye of a 17-year-old male submacular hemorrhage (SMH) patient secondary to traumatic retinopathy. **a** Fundus photograph showed large SMH (red arrow); **b** Swept-source optical coherence tomography (SS-OCT) showed large SMH (red arrow); **c** Postoperative fundus photograph showed complete absorption of the SMH (red arrow) and the perfluoropropane (C3F8) tamponade; **d** Postoperative SS-OCT showed complete absorption of the SMH (red arrow) and the injection site of the 41-gauge subretinal needle (green arrow)

The main outcome measures included BCVA, SMH absorption, and perioperative complications. The Snellen BCVA was then converted to the logarithm of the minimum angle of resolution (logMAR) equivalents for statistical analysis [18]. The vision of no light perception (NLP), light perception (LP), hand movement (HM), and finger counting (FC) was designated as 2.90, 2.60, 2.30, and 1.85 logMAR [19], respectively. Also, the postoperative BCVA was compared to the preoperative BCVA, and then the prognosis of BCVA was categorized into “improved”, “stable”, and “worsened”. SMH absorption was grouped into “complete absorption”, “partial absorption”, and “no absorption”. “Complete absorption” was defined as the absence of blood in the foveal area on the postoperative OCT or FP (shown in Fig. 2); “partial absorption” was defined as a reduction in the amount of subfoveal blood but still with remaining blood or fibrosis in the foveal area after the surgery (shown in Fig. 1c, d); “no absorption” was defined as no reduction in the amount of subfoveal blood.

### Statistical analysis

All data were collected and evaluated by two retinal specialists (XYZ and QZ). The measurement data were collected three times and the average values were used for evaluation. For the classification data and descriptive data, the evaluation was made separately, and the Cohen’s Kappa test was used to assess the inter-rater agreement. Continuous variables were summarized as mean ± standard deviation (SD), and categorical data were presented as frequency (percentages). Paired t-test was used to compare BCVA between baseline and postoperative time points. Multiple linear regression analysis was used to investigate the correlation between clinical characteristics and the postoperative BCVA, and univariate binary logistic regression analysis was conducted ﻿using various baseline parameters to identify correlated factors for postoperative SMH or VH occurrence. All statistical analyses were performed with Stata SE 12.0 software (StataCorp, College Station, TX, USA), and a *P* value of less than 0.05 was considered statistically significant.

## Results

### Baseline characteristics

A final total of 18 eyes of 18 patients who underwent PPV combined with subretinal injection of rt-PA using the 3D heads-up system were included in this study. The data collected are shown in Tables 1 and 2. Among all included patients, 11 (61.11%) were male and 6 (38.89%) were female, with a mean age of 62.06 ± 17.73 years. The most common primary disease of SMH was PCV (10, 55.56%), followed by RAM (5, 27.78%), traumatic retinopathy (2, 11.11%), and nAMD (1, 5.56%). Intravitreal injections of anti-vascular endothelium growth factor (anti-VEGF) agents were used in 9 (50.00%) eyes preoperatively. Besides, focal photocoagulation, photodynamic therapy, and retrobulbar glucocorticoid each were performed in 1 (5.56%) eye preoperatively. The mean preoperative BCVA was 1.82 ± 0.62 logMAR with a range from 2.60 logMAR (LP) to 0.92 logMAR (Snellen BCVA: 0.3). In all cases, the hemorrhage involved the foveal center (18, 100.00%). The mean GLD and height of SMH were 6538.17 ± 2533.11 μm and 937.36 ± 420.21 μm, respectively. Cataract was found preoperatively in 12 (66.67%) eyes, followed by VH in 8 (44.44%) eyes, pseudophakia in 2 (11.11%) eyes, hemorrhagic PED in 2 (11.11%) eyes, and hemorrhagic RD in 1 (5.56%) eye.

**Table 1:** Clinical data and prognosis of the included submacular hemorrhage patients

Case/age (years)/sex/side/disease	Treatment before SMH	GLD /height (μm)	Foveal involvement	Special feature	Interval (days)	Surgery details	Preoperative BCVA (logMAR)	Final BCVA (logMAR)	Follow-up (days)	SMH prognosis	Visual prognosis	Complications
1/80/M/OD/RAM	IVR × 1	5609/1200	Yes	Large SMH, preretinal hemorrhage and retinal break	14	PPV + ICG + ILM peeling + rt-PA + air-fluid exchange + endolaser + silicone	2.30	1.30	100	Complete absorption	Improved	No
2/17/M/OS/traumatic retinopathy	Retrobulbar GC × 2	3559/491	Yes	Large SMH	11	PPV + ICG + ILM peeling + rt-PA + 14% C3F8	1.00	0.10	157	Complete absorption	Improved	No
3/80/M/OS/PCV	IVA × 1	NA/NA	Yes	VH, large SMH, hemorrhagic PED and severe cataract	180	Phaco + PPV + ICG + ILM peeling + rt-PA + IOL + 14% C3F8	2.60	2.60	123	Recurrent VH, SMH and hyphema	Stable	Recurrent VH, SMH, hyphema and secondary PPV with silicone tamponade
4/20/M/OD/traumatic retinopathy	No treatment	2985/529	Yes	Large SMH	12	PPV + ICG + ILM peeling + rt-PA + 14% C3F8	1.85	1.00	96	Partial absorption	Improved	No
5/68/M/OS/RAM	FPC × 1	NA/NA	Yes	Large SMH	7	Phaco + PPV + ICG + ILM peeling + rt-PA + IOL + 14% C3F8	1.00	0.40	184	Complete absorption	Improved	No
6/68/F/OD/PCV	No treatment	8742/715	Yes	Large SMH	7	PPV + ICG + ILM peeling + rt-PA + 14% C3F8 + IVA × 1	1.00	0.52	90	Complete absorption	Improved	No
7/66/F/OD/nAMD	PDT × 1 + IVC × 7	6821/553	Yes	Large SMH	30	PPV + ICG + ILM peeling + rt-PA + 14% C3F8 + IVA × 1	0.92	0.40	99	Complete absorption	Improved	No
8/65/M/OD/RAM	No treatment	7786/1069	Yes	Large SMH	20	Phaco + PPV + ICG + ILM peeling + rt-PA + IOL + 14% C3F8	1.52	1.00	131	Complete absorption	Improved	Delayed RD and secondary PPV
9/73/M/OD/PCV	IVA × 6	8755/728	Yes	VH, large SMH and hemorrhagic RD	15	Phaco + PPV + ICG + ILM peeling + rt-PA + IOL + 14% C3F8	2.30	0.92	143	Complete absorption	Improved	No
10/69/M/OD/PCV	IVA × 3	7364/1884	Yes	VH and large SMH	90	Phaco + PPV + ICG + ILM peeling + rt-PA + IOL + 14% C3F8 + IVA × 1	1.70	1.70	95	No absorption	Stable	No
11/72/M/OD/PCV	No treatment	11,860/1340	Yes	Large SMH	10	Phaco + PPV + ICG + ILM peeling + rt-PA + IOL + 14% C3F8	1.00	0.82	198	Complete absorption	Improved	No
12/70/F/OD/PCV	IVC × 1	NA/NA	Yes	VH, large SMH and hemorrhagic PED	14	PPV + ICG + ILM peeling + rt-PA + 14% C3F8	2.30	2.30	131	Recurrent VH, SMH and hyphema	Stable	Recurrent VH, SMH, hyphema and secondary PPV with silicone tamponade
13/69/M/OD/PCV	IVA × 12	5319/NA	Yes	Large SMH	90	PPV + ICG + ILM peeling + rt-PA + endolaser + 14% C3F8 + IVA × 1	2.30	2.60	148	Partial absorption	Worsened	RD and secondary PPV with silicone tamponade
14/67/M/OD/PCV	IVA × 3	NA/NA	Yes	VH and large SMH	60	Phaco + PPV + ICG + ILM peeling + rt-PA + IOL + 14% C3F8	2.60	2.30	116	Partial absorption	Improved	No
15/45/F/OD/PCV	IVC × 3	NA/NA	Yes	VH and large SMH	30	PPV + ICG + ILM peeling + rt-PA + 14% C3F8	2.30	0.60	125	Partial absorption	Improved	No
16/71/F/OS/RAM	No treatment	4340/825	Yes	Large SMH	60	Phaco + PPV + ICG + ILM peeling + rt-PA + IOL + 14% C3F8	2.00	0.82	99	Partial absorption	Improved	No
17/60/F/OD/PCV	No treatment	NA/NA	Yes	VH and large SMH	15	PPV + ICG + ILM peeling + rt-PA + 14% C3F8	2.30	1.00	99	Partial absorption	Improved	No
18/57/F/OS/RAM	No treatment	5318/977	Yes	VH and large SMH	24	PPV + rt-PA + TA + ICG + air-fluid exchange + 10% C3F8	2.30	0.22	225	Complete absorption	Improved	No

*anti-VEGF* = anti-vascular endothelium growth factor; *BCVA* = best-corrected visual acuity; *C3F8* = perfluoropropane; *F* = female; *FPC* = focal photocoagulation; *GC* = glucocorticoid; *GLD* = greatest linear dimension; *ICG* = indocyanine green; *ILM* = internal limiting membrane; *IVA* = intravitreal aflibercept; *IVC* = intravitreal conbercept; *IVR* = intravitreal ranibizumab; *IOL* = intraocular lens; *M* = male; *NA* = not applicable; *nAMD* = neovascular age-related macular degeneration; *PCV* = polypoidal choroidal vasculopathy; *PED* = pigment epithelial detachment; *PPV* = pars plana vitrectomy; *RAM* = retinal arterial microaneurysm; *rt-PA* = recombinant tissue plasminogen activator; *RD* = retinal detachment; *SMH* = submacular hemorrhage; *TA* = triamcinolone acetonide; *VH* = vitreous hemorrhage

**Table 2:** Baseline information and clinical characteristics of SMH patients

Basic information and clinical characteristics	SMH (n = 18 eyes of 18 patients)
Age, years (mean ± SD)	62.06 ± 17.73
Sex (male), n (%)	11 (61.11%)
Primary disease, eyes (%)	
PCV	10 (55.56%)
nAMD	1 (5.56%)
RAM	5 (27.78%)
Traumatic retinopathy	2 (11.11%)
Preoperative BCVA, logMAR (mean ± SD)	1.85 ± 0.62
Foveal involvement, eyes (%)	18 (100.00%)
GLD of SMH, μm (mean ± SD)	6538.17 ± 2533.11
Height of SMH, μm (mean ± SD)	937.36 ± 420.21
Comorbidity, eyes (%)	
Cataract	12 (66.67%)
Pseudophakia	2 (11.11%)
VH	8 (44.44%)
RD	1 (5.56%)
Hemorrhagic PED	2 (11.11%)
Interval from disease onset to surgery, days (mean ± SD)	38.28 ± 44.40
Surgery details	
Duration of ILM peeling, min (mean ± SD)	5.54 ± 3.39
Duration of rt-PA injection, min (mean ± SD)	3.14 ± 1.25
C3F8 tamponade, eyes (%)	17 (94.44%)
Silicone oil tamponade, eyes (%)	1 (5.56%)
Combined with cataract surgery, eyes (%)	8 (44.44%)
Combined with anti-VEGF treatment, eyes (%)	4 (22.22%)
Intraoperative iatrogenic retinal breaks, eyes (%)	0 (0%)
Postoperative ocular status, eyes (%)	
With complications requiring additional surgery	4 (22.22%)
SMH complete absorption	9 (50.00%)
SMH partial absorption	6 (33.33%)
No absorption	1 (5.56%)
Total number of additional PPV, n (%)	4 (22.22%)
Final BCVA, logMAR (mean ± SD)	1.14 ± 0.82^*^
Postoperative visual prognosis, eyes (%)	
Improved	14 (77.78%)
Stable	3 (16.67%)
Worsened	1 (5.56%)
Complications, eyes (%)	
Recurrent SMH or VH	2 (11.11%)
RD	2 (11.11%)
Endophthalmitis	0 (0.00%)
Follow-up period, days (mean ± SD)	122.17 ± 48.83

*anti-VEGF* = anti-vascular endothelium growth factor; *BCVA* = best-corrected visual acuity; *C3F8* = perfluoropropane; *GLD* = greatest linear dimension; *ILM* = internal limiting membrane; *nAMD* = neovascular age-related macular degeneration; *PCV* = polypoidal choroidal vasculopathy; *PED* = pigment epithelial detachment; *PPV* = pars plana vitrectomy; *RAM* = retinal arterial microaneurysm; *rt-PA* = recombinant tissue plasminogen activator; *RD* = retinal detachment; *SD* = standard deviation; *SMH* = submacular hemorrhage; *VH* = vitreous hemorrhage

^*^The final BCVA was significantly improved compared with preoperative BCVA (*P* < 0.05)

### Surgical characteristics

The mean interval from SMH onset to the time of the surgery was 38.28 ± 44.40 days, with the longest interval of 180 days (shown in Table 2). The surgical step of rt-PA injection lasted for 3.14 ± 1.25 min, and the ILM peeling lasted for 5.54 ± 3.39 min. C3F8 tamponade was performed in 17 (94.44%) eyes, while silicone oil tamponade was performed in 1 (5.56%) eye. Subretinal injections of rt-PA in 8 (44.44%) eyes were combined with cataract surgery, and 4 (22.22%) eyes were combined with intravitreal injection of anti-VEGF agent intraoperatively. No iatrogenic retinal break was found intraoperatively.

### Effectiveness and complications

After an average follow-up time of 131.06 ± 38.96 days with a range of 90 to 225 days, the BCVA was significantly improved to 1.14 ± 0.82 logMAR postoperatively (*P* < 0.05) with a range from 2.60 logMAR (LP) to 0.10 logMAR (Snellen BCVA: 0.8) (shown in Table 2). The postoperative BCVA was improved in 14 (77.78%) eyes, stable in 3 (16.67%) eyes, and worsened in 1 (5.56%) eye. The SMH was completely absorbed in 9 (50.00%) eyes, partially absorbed in 6 (33.33%) eyes, and not absorbed in 1 (5.56%) eye. In multiple linear regression analysis, preoperative BCVA was significantly correlated with postoperative BCVA (*P* < 0.05).

Severe complications were noticed in 4 (22.22%) eyes, including 2 (11.11%) with recurrent SMH or VH and 2 (11.11%) with RD occurrence. All these 4 (22.22%) eyes underwent additional PPV to manage these severe complications. The univariate binary logistic regression analysis identified the comorbidity of hemorrhagic PED as a factor associated with SMH and VH recurrence (*P* < 0.05).

## Discussion

To the best of our knowledge, this is the first study to evaluate the application of the 3D heads-up system in subretinal rt-PA injection and PPV for SMH. Most of the included SMH patients were secondary to PCV, which is an exudative maculopathy occasionally causing massive SMH. Postoperatively, complete or partial SMH absorption was achieved in 83.33% of patients, with BCVA significantly improved from preoperative status. Our multiple linear regression analysis suggested that preoperative BCVA might be a prognostic factor for postoperative BCVA. No iatrogenic retinal break occurred during the surgery. However, postoperative SMH and/or VH recurrence and RD occurrence each occurred in two patients and required additional PPVs. The SMH and/or VH recurrence was found to be associated with the preoperative presence of hemorrhagic PED.

### Surgical procedures

The subretinal injection could cause possible mechanical damage to the retinal nerve fiber layer, RPE, and choroid. Multiple complications with subretinal injections have been reported previously, such as retinal and/or RPE tears, choroidal neovascularization, and so on [20]. Considering that, minimizing the collateral damage of the injection procedure is extremely important. The ILM, the basement membrane of Müller cells, is composed of multiple extracellular matrix proteins, and its stiffness increases with age [21]. A recent study reported that the ILM is the greatest source of physical resistance to subretinal injection [22]. Okanouchi et al. [23] observed that ILM peeling could halve the pressure needed for needle penetration and keep the injection pressure relatively low. Therefore, we removed the ILM at the injection site, hopefully reducing mechanical damage to the surrounding tissues and decreasing the risk of subretinal injection-related complications.

### Effectiveness

The management of SMH has evolved greatly in the past 30 years and a variety of therapeutic approaches have been developed, including transplantation of RPE or a choroidal patch, photodynamic therapy, intravitreal injection of anti-VEGF agents, PPV with submacular surgery and displacement of SMH with expansile gas [24]. Recently, intravitreal or subretinal rt-PA injection combined with PPV and vitreous tamponade was shown to be comparably effective in the treatment of SMH [25, 26]. In our study, the percentage of complete or partial SMH absorption was 83.33%, similar to the previous study [25]. Kitagawa et al. [27] previously reported that the baseline BCVA was the factor affecting the postoperative BCVA, which was also found in our study. Other factors found to be associated with the final BCVA included the GLD of SMH [28], preoperative detectable ellipsoid layers, and preoperative SMH height less than 400 μm [29]. Future studies are needed to determine the factors that can predict a good visual prognosis of SMH after surgery.

### Complications

Here, we observed the occurrence of VH, SMH, and RD as postoperative complications, which were also reported in previous studies [5, 6, 30]. Based on our surgical experience, two possible explanations were proposed for the occurrence of these complications. First, the thrombolytic property of rt-PA causes tissue lysis and induces retinal toxicity, which may further increase the fragility of the retina. Second, even though the 41-gauge subretinal needle with an outer diameter of only 80 μm was used in this study, the risk of bleeding into the vitreous through the retinal hole created with the needle after the submacular injection still existed. To minimize retina damage and surgical complications, Kadonosono et al. [31] used the 47-gauge (50 μm) microneedle in their surgical procedures.

Saito-Uchida et al. [5] reported SMH and/or VH recurrence rate of 73% in 11 eyes after subretinal injection of rt-PA using the TM system and 5 (45%) more surgeries were needed to manage these complications. Other studies reported that 9 (60%) of 15 eyes and 10 (41.6%) of 24 eyes required at least one additional surgery for managing the postoperative complications of SMH, VH, or RD [32, 33]. The rate of postoperative complications in these aforementioned studies was both much higher than that observed in our study. This difference might be explained by the application of the 3D heads-up system. The 3D surgical system has been reported to have multiple advantages including a wider visual field and higher magnification performance compared with the TM system [12, 13]. Our previous study further indicated that the 3D heads-up system was associated with a significantly shorter duration of delicate surgical steps, like ILM peeling, than the TM system [14]. Besides, previous studies also reported the occurrence of iatrogenic circumscribed semicircular rip in the RPE and macular hole caused by the inadvertent injection of the rt-PA [31, 34, 35], which may be avoided using the 3D heads-up system with a higher magnification. In our study, no iatrogenic retinal break, macular hole, RPE tear, or other intraoperative complication was noticed. These findings suggest that the 3D heads-up system can enable a more precise and safer subretinal rt-PA injection and help reduce perioperative complications.

Two PCV eyes with SMH and hemorrhagic PED (case 3 and case 12, see Table 1) were complicated with postoperative SMH and VH recurrence, which further led to hyphema. Additional PPVs with silicone tamponade were performed to clean the hemorrhage and prevent further complications. For case 3, considering his advanced age and multiple comorbidities including heart failure, the silicone oil was not removed postoperatively after a full discussion with the patient and his family. For case 12, PPV combined with silicone oil tamponade was performed, and her BCVA was maintained at HM with no hemorrhage recurrence after the removal of silicone oil postoperatively. Our previous PPV experience on more than 100 PCV cases with VH suggested that the pros and cons should be weighed seriously before conducting a retinotomy to drain these hemorrhages and iatrogenic retinal break should also be avoided [36]. In this study, univariate binary logistic regression analysis also confirmed hemorrhagic PED as a factor associated with SMH and VH recurrence. Therefore, we suggest that subretinal injection of rt-PA should be used with caution for PCV patients with hemorrhagic PED and massive SMH involving the whole posterior pole, and silicone oil tamponade may be a preferred alternative as it could prevent recurrent SMH and/or VH. The reasons are as follows: after the pharmacological lysis of the SMH using rt-PA, massive SMH leaks into the subretinal space, rapidly decreasing the subretinal pressure; the decrease of pressure combined with the high bleeding tendency of PCV lesions will trigger rebleeding immediately and induces massive SMH and VH, which becomes extremely difficult to manage.

### Limitations

Several limitations of our study should be noted. First, no control group using the TM system was assigned. Further studies comparing the subretinal injection of rt-PA using the TM system and the 3D heads-up system are needed to highlight the advantages of 3D surgery. Second, the sample size was small. However, to the best of our knowledge, this is the largest study to report the outcomes of subretinal injection of rt-PA using the 3D heads-up system. Third, the follow-up period may be short to evaluate the visual outcomes and complications, and a longer follow-up period is needed. Finally, we evaluated only the visual outcomes and complications, while the retinal sensitivity test determined by microperimetry should also be performed.

## Conclusions

The 3D heads-up system assisted-PPV and subretinal injection of rt-PA were efficacious in eliminating SMH and improving visual prognosis with satisfactory safety. However, for PCV patients with hemorrhagic PED and massive SMH, using silicone tamponade should be considered first as it has been shown to prevent recurrent SMH and VH.

## Data Availability

The datasets used and/or analyzed during the current study are available from the corresponding author on reasonable request.

## Abbreviations

Anti-VEGF: Anti-vascular endothelium growth factor
BCVA: Best-corrected visual acuity
C3F8: Perfluoropropane
FC: Finger counting
FP: Fundus photograph
GLD: Greatest linear dimension
HM: Hand movement
ICG: Indocyanine green
ILM: Internal limiting membrane
IOP: Intraocular pressure
logMAR: Logarithm of the minimum angle of resolution
LP: Light perception
nAMD: Neovascular age-related macular degeneration
NLP: No light perception
OCT: Optical coherence tomography
PCV: Polypoidal choroidal vasculopathy
PED: Pigment epithelial detachment
PPV: Pars plana vitrectomy
RAM: Retinal arterial microaneurysm
RPE: Retinal pigment epithelium
rt-PA: Recombinant tissue plasminogen activator
RD: Retinal detachment
SD: Standard deviation
SS-OCT: Swept-source optical coherence tomography
SMH: Submacular hemorrhage
TM: Traditional microscopic
VH: Vitreous hemorrhage
3D: Three-dimensional
